# A comparative study of traditional Chinese mind-body therapies on sleep quality, mental health, and quality of life in patients with sleep disorders: a network meta-analysis based on randomized controlled trials

**DOI:** 10.3389/fpsyt.2026.1921194

**Published:** 2026-09-18

**Authors:** Xianzhou An

**Affiliations:** Luoyang Normal University, Luoyang, China

**Keywords:** mental health, meta-analysis, mind-body exercise, quality of life, quality of sleep, sleep disorder

## Abstract

**Background:**

Sleep disorders are a common clinical condition characterized primarily by difficulty falling asleep, trouble maintaining sleep, and impaired daytime functioning, with a high prevalence among adults. This condition can lead to a persistent decline in sleep quality, trigger mental health issues such as anxiety and depression, and ultimately reduce overall quality of life. Traditional Chinese mind-body exercise therapies are considered potentially effective non-pharmacological interventions due to their low risk, high accessibility, and dual mind-body regulation effects. However, the relative efficacy of different exercise therapies on sleep quality, mental health, and quality of life in patients with sleep disorders remains unclear. This study employed a web-based meta-analysis to systematically evaluate the effects of three interventions—Tai Chi, Baduanjin, and Tai Chi combined with transcranial magnetic stimulation—on three outcome measures and to rank their efficacy, aiming to provide evidence-based support for clinical decision-making.

**Methods:**

This study conducted a systematic search of the PubMed, Embase, Web of Science, Cochrane, EBSCO, and CNKI databases and included 18 randomized controlled trials that met the inclusion criteria. Outcome measures included sleep quality, mental health, and quality of life. A network meta-analysis using a random-effects model was performed to calculate standardized mean differences and 95% confidence intervals, and the cumulative ranking probability curve area was used to rank the efficacy of the interventions.

**Result:**

This study included a total of 18 randomized controlled trials. Regarding sleep quality, TCCWTMS yielded the best results (SMD = −1.15, 95% CI [−2.07, −0.22]). Regarding mental health, only the Tai Chi intervention achieved statistical significance (SMD = 0.64, 95% CI [0.07, 1.21], P = 0.03); although Baduanjin ranked first in the SUCRA ranking (92.6%), pairwise comparisons did not reach statistical significance (P = 0.07). Regarding quality of life, both Tai Chi (SMD = -0.47, 95% CI [-0.71, -0.24], P < 0.0001) and Baduanjin (SMD = -0.57, 95% CI [-0.91, -0.23], P = 0.001) were significantly effective. There were significant heterogeneous differences in the efficacy of the three interventions across different outcome measures.

**Conclusion:**

This study indicates that the TCCWTMS is the most effective intervention for improving sleep quality in patients with sleep disorders. Regarding quality of life, both Baduanjin and Tai Chi demonstrated significant effects, with Baduanjin showing superior efficacy. Regarding mental health, the current evidence is insufficient to determine the optimal intervention strategy. There are significant differences in the efficacy of the three interventions across various outcome measures; clinical decisions can be made by selecting the appropriate intervention based on the patient’s score needs.

**Systematic review registration:**

https://www.crd.york.ac.uk/PROSPERO/view/CRD420261415202, identifier CRD420261415202.

## 1 Introduction

Sleep disorders are a clinical syndrome characterized primarily by difficulty falling asleep, trouble maintaining sleep, and impaired daytime functioning, and they have a high prevalence among adults. Epidemiological studies indicate that approximately 59% of the global population experiences sleep problems to varying degrees, with about 28% of individuals sleeping less than 6 hours per night (1). Chronic sleep disorders can lead to fragmented sleep architecture and reduced slow-wave sleep, directly impairing sleep quality (2). At the same time, sleep deprivation can trigger emotional disturbances such as anxiety and depression, jeopardizing mental health (3). This further limits patients’ social participation and ability to perform daily activities, causing lasting negative impacts on their overall quality of life (4). Given the significant impact of sleep disorders on public health, exploring safe, effective, and sustainable non-pharmacological intervention strategies has become a major research focus in the field of sleep medicine (5).

Improvement in sleep quality is the primary outcome measure for evaluating the effectiveness of sleep disorder interventions, directly reflecting restorative changes in patients’ nocturnal sleep (6). Mental health involves improvements in emotional states such as anxiety and depression, which frequently co-occur with sleep disorders and are mutually causative (7, 8). Quality of life serves as a comprehensive outcome measure, encompassing multiple dimensions such as social interaction and subjective well-being, and reflecting the ultimate impact of interventions on patients’ overall health status (9, 10). Together, these three indicators form the core framework for assessing clinical outcomes in patients with sleep disorders across different levels, providing a comprehensive evaluation of the clinical value of interventions.

Currently, the standard treatment for sleep disorders primarily involves benzodiazepines and non-benzodiazepine hypnotics. However, long-term use of these medications can lead to drug dependence, increased tolerance, and rebound insomnia upon discontinuation (11). Although cognitive behavioral therapy is recommended as a first-line non-pharmacological treatment, adherence to it is limited among the elderly (12), and the absolute magnitude of improvement in sleep parameters is relatively modest (13). Against this backdrop, traditional Chinese mind-body exercise therapies have garnered widespread attention due to their low risk, high accessibility, and dual mind-body regulation characteristics. Tai Chi and Baduanjin, as typical examples, are believed to reduce sympathetic nervous system excitability and promote relaxation through integrated training involving slow movements, breath control, and mental focus (14, 15). In recent years, studies have also explored the synergistic effects of combined Tai Chi combined with transcranial magnetic stimulation. However, existing reviews have primarily focused on paired comparisons between single therapies and conventional controls, and there remains a lack of head-to-head comparisons of different intervention types and combined regimens within a network meta-analysis framework (16). In particular, differences in efficacy across various outcome measures—such as sleep quality, mental health, and quality of life—remain to be clarified.

Therefore, this study conducted a network meta-analysis based on randomized controlled trials to systematically evaluate the relative efficacy of three interventions—TC, BDJ, and TCCWTMS—on sleep quality, mental health, and quality of life in patients with sleep disorders (17). By providing a quantitative ranking of the efficacy of different interventions, this study aims to provide evidence-based guidance for clinical decision-makers and patients in selecting the optimal treatment regimen (18, 19).

## 2 Methods

We report this meta-analysis in accordance with the 2020 PRISMA Statement. The study protocol for this systematic review has been registered with PROSPERO. Registration number: CRD420261415202. As this is a review study, we did not need to seek approval of the study protocol from an ethics committee or institutional review board, nor did we need to obtain informed consent from study participants.

### 2.1 Data sources

We conducted a systematic search of databases including PubMed, Embase, Web of Science, Cochrane, EBSCO, and CNKI. Two researchers independently screened the relevant studies. MeSH terms were used to search PubMed and Cochrane; Emtree terms were used to search Embase; and a combination of subject headings and free-text keywords was used to search CNKI. In addition, we manually reviewed the reference lists of relevant literature to identify potentially eligible studies. The search period spanned from 2004 to 2025, and only human studies published in Chinese or English were included. Chinese studies were limited to those published in core journals.

This study followed the PICOS framework for evidence-based medicine: (P) Population: patients with sleep disorders and insomnia; (I) Intervention: Tai Chi, Baduanjin, or Tai Chi combined with transcranial magnetic stimulation; (C) Control group: no intervention or routine treatment; (O) Outcomes: quality of sleep; mental health; quality of life; (S) Study design: randomized controlled trials.

### 2.2 Study selection

We conducted a systematic literature search using PubMed and the Cochrane Database, employing the Medical Subject Headings (MeSH) terminology. To ensure the comprehensiveness and accuracy of the search, we manually screened the reference lists of included articles to identify other potentially eligible studies, thereby achieving comprehensive coverage of the literature on traditional Chinese mind-body exercise therapies and sleep disorders. Following the initial search, we rigorously screened the identified literature. First, we used EndNote software to automatically identify and remove duplicate records resulting from overlapping search strategies or databases. Subsequently, we manually reviewed titles and abstracts to further exclude duplicates not identified by the automated process, thereby ensuring the uniqueness and representativeness of the literature. The remaining studies underwent a more rigorous review, primarily excluding the following types: studies involving non-sleep disorders, studies that did not assess relevant outcome measures, and studies that did not use traditional Chinese mind-body exercises as an intervention. Additionally, review articles, conference abstracts, animal studies, research protocols, case reports, retrospective studies, and book chapters were excluded. Such literature typically lacks raw data or sufficient scientific rigor, making it difficult to conduct effective analyses of intervention effects and draw valid conclusions. The aforementioned rigorous literature screening criteria aim to ensure the high quality of the ultimately included studies, providing reliable evidence to support this research and thereby enhancing its overall scientific rigor and credibility.

### 2.3 Eligibility criteria

We included randomized clinical trials targeting patients with sleep disorders; all studies were conducted specifically for this patient population.

Studies meeting the inclusion criteria must satisfy the following conditions: (1) be randomized controlled trials (RCTs); (2) involve adult patients presenting with clinically diagnosed or established sleep disturbances (e.g., insomnia symptoms, poor sleep quality) as verified by validated screening tools (such as the Pittsburgh Sleep Quality Index, PSQI) or clinical diagnostic criteria; (3) provide complete records of outcome measures; (4) The experimental group received traditional Chinese mind-body exercise therapies (including TC, BDJ, TCCWTMS, etc.), while the control group received no intervention or only conventional treatment; (5) At least the following indicators were assessed: For sleep quality (QOS), the Pittsburgh Sleep Quality Index (PSQI) was the primary assessment tool, with supplementary use of the Insomnia Severity Index (ISI) and Epworth Sleepiness Scale (ESS). For mental health (MH), the included studies utilized the Hamilton Anxiety Scale (HAMA), Hamilton Depression Scale (HAMD), and the Hospital Anxiety and Depression Scale (HADS-D). For quality of life (QOL), assessments were conducted using the Short Form-12 (SF-12), Short Form-36 (SF-36). All outcomes were treated as continuous variables for the network meta-analysis. Exclusion criteria included: (1) non-randomized controlled trials; (2) animal studies, review articles, conference proceedings, case reports, letters to the editor, or duplicate publications; (3) articles for which the full text was unavailable; (4) studies with incomplete data or from which key indicators could not be extracted; (5) studies that did not report the relevant indicators of interest in this review; (6) patients with other syndromes.

### 2.4 Data collection

Two researchers imported the collected literature into EndNote 20 software and conducted an initial screening based on a predefined strategy. First, duplicate records were removed. Subsequently, an initial screening was completed by reviewing the titles and abstracts. Next, the full texts were carefully reviewed against the inclusion and exclusion criteria. Subsequently, the two researchers cross-checked the screening results. If consensus was reached, the study was included; if a disagreement arose, a third researcher was consulted. The final decision on inclusion was determined through discussion and consensus.

For trials meeting the inclusion criteria, two trained researchers independently extracted data from the relevant literature using standardized data extraction forms and assessed the risk of bias. The extracted data primarily included: (1) study background information (first author, year of publication, country, etc.); (2) demographic characteristics of participants (number of participants, age, gender in the experimental and control groups); (3) details of the intervention (type, duration, and frequency); (4) outcome measures (mean and standard deviation; selected primary outcomes included sleep quality, mental health, and quality of life). For studies that presented results only in graphical form without numerical summaries, we used a validated graph digitization tool (GetData 2.22) to extract numerical data for analysis. When necessary, we contacted the authors of the articles to obtain information.

### 2.5 Data risk of bias of the systematic review

Using the Cochrane Risk of Bias Assessment Tool (Version 5.1; Higgins et al., 2011), two researchers independently assessed the risk of bias (ROB) across seven domains for all studies meeting the inclusion criteria: random sequence generation, allocation concealment, participant and researcher blinding, outcome assessor blinding, incomplete outcome data, selective reporting of results, and other potential sources of bias. The assessment was conducted using Review Manager 5.3 software (The Nordic Cochrane Centre, Denmark), with each domain classified as “low risk,” “high risk,” or “unclear.” Based on these assessments, the overall risk of bias for each study was categorized as follows: (1) Low risk of bias: No domains rated as high risk, and fewer than three domains rated as “unclear”; (2) Moderate risk of bias: Only one dimension was rated as high risk; or no dimensions were rated as high risk, but more than three dimensions were rated as “unclear”; (3) High risk of bias: Studies that did not meet either of the above two criteria were classified into this category.

We applied the Grading of Recommendations Assessment, Development and Evaluation (GRADE) framework to evaluate the certainty of evidence for each outcome. Two reviewers independently assessed five key domains—including risk of bias, inconsistency, indirectness, imprecision, and publication bias—with discrepancies settled by consensus. Evidence certainty was ultimately classified as high, moderate, low, or very low.

### 2.6 Statistical analysis

All statistical analyses were performed using STATA 17.0 software (Stata Software LLC, University City, Texas, USA) and R software (Version 4.5.2). Since all outcome measures were continuous variables, we used standardized mean differences (SMDs) with 95% confidence intervals (CIs) as the summary effect measures. A frequentist random-effects model was employed to account for heterogeneity across studies. For the synthesis of direct pairwise comparisons, we utilized post-treatment mean values and standard deviations (SDs) to calculate the effect sizes. In cases where studies only reported change scores (mean change and SD of change) without providing post-treatment data, we prioritized the change scores as the basis for analysis, as this approach minimizes the influence of baseline imbalances. When a study reported neither the SD of change nor the post-treatment SD, and no available p-value or t-statistic for imputation, we contacted the corresponding authors to request the missing data. If the original data were not obtainable, we excluded the study from the specific outcome synthesis to avoid introducing statistical bias from arbitrary imputation methods. Heterogeneity was assessed using the I²statistic and Cochran’s Q test, with I²>50% or a significant Q-test (P<0.10) indicating substantial heterogeneity. To test the transitivity assumption underlying the network meta-analysis, we visually compared the distribution of key clinical and methodological characteristics (such as age, intervention duration, and baseline disease severity) across the included studies. Local inconsistency between direct and indirect evidence was evaluated using the node-splitting method, which examines whether the effect estimates derived from direct comparisons are statistically consistent with those obtained from indirect evidence. A non-significant result from the node-splitting test indicates no significant inconsistency within the network. Network relationships were visualized through network contribution diagrams, where node size and edge thickness corresponded to the number of studies for each direct comparison. Publication bias was assessed using adjusted comparison funnel plots. Finally, the cumulative ranking probability curve area (SUCRA) was calculated to determine the probability of each intervention being the optimal treatment.

## 3 Result

### 3.1 Study selection

The PRISMA flow diagram illustrates the study selection process. The initial search identified a total of 2,710 records from PubMed (n = 902), Web of Science (n = 703), the Cochrane Library (n = 211), CNKI (n = 32), Embase (n = 521), and EBSCO (n = 341). After removing 1,011 duplicate records and 976 records excluded for other reasons, 723 unique records remained for title and abstract screening. Following this stage, 231 records were excluded, leaving 492 articles for full-text review. Of these, the full text could not be obtained for 0 articles, and 492 articles were finally assessed for eligibility. After full-text assessment, 474 articles were excluded for the following reasons: inappropriate study design (n = 230), irrelevant topic (n = 183), and ineligible outcome measures (n = 61). No additional eligible studies were identified through citation searching or organizational websites. In total, the initial search identified 18 newly eligible studies, which resulted in a final total of 18 included studies. The detailed study selection process is presented in **Figure 1**.

**Figure 1:**
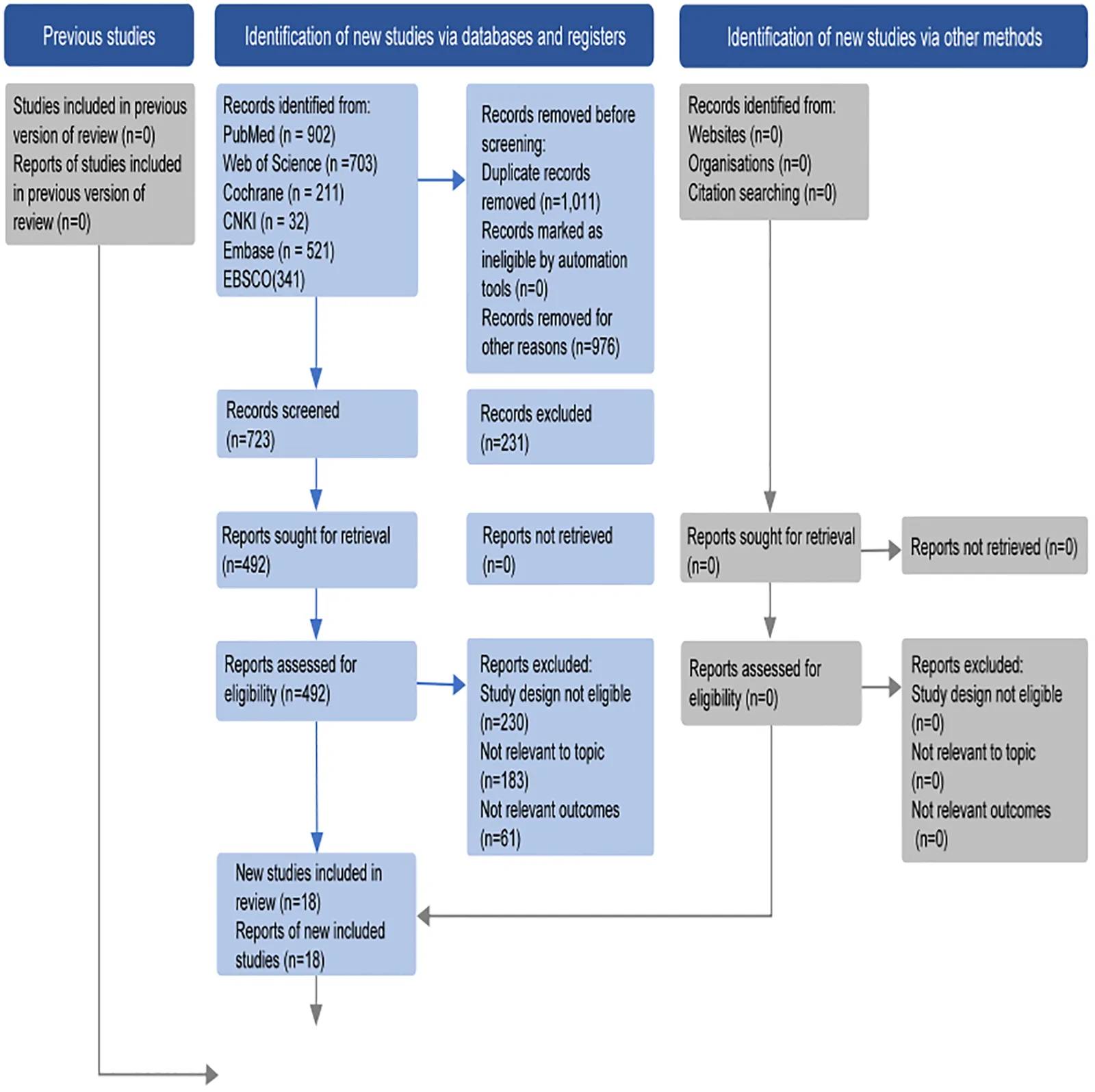
Literature search flowchart.

### 3.2 Literature characterization

A total of 18 studies were ultimately included; a detailed list of their baseline characteristics is provided in **Appendix 1**. This systematic review and network meta-analysis covered studies published between 2004 and 2025 in the United States, Iran, Vietnam, China, Hong Kong, China, and Japan. The experimental groups included 142 patients who received the TC intervention, 90 patients who received the BDJ intervention, and 93 patients who received the TCCWTMS intervention. The control groups included 617 participants aged between 45 and 78 years, for a total of approximately 850 subjects. Appendix 1 provides a detailed list of the characteristics of the included studies. Across 18 randomized controlled trials, we compared the effects of the three interventions (TC, BDJ, and TCCWTMS) with those of the control group. TC was the most commonly used intervention, followed by BDJ. Each intervention session lasted between 25 and 80 minutes. The average duration of the exercise interventions was 12 weeks (range: 12 days to 24 weeks), with an average of 3.2 exercise sessions per week.

### 3.3 Risk of bias assessment

**Appendix 1** and **Figure 2** provide detailed results of the risk of bias assessments for each study. Among these 18 studies, all employed a randomized allocation design: 14 described the randomization method in detail, and 7 discussed allocation concealment strategies; 8 reported implementation blinding, and 11 reported outcome assessment blinding; all 18 studies showed a low risk of selective reporting; no other sources of bias were identified in any of the studies. Overall, the risk of bias (ROB) for these 13 studies was assessed as low.

**Figure 2:**
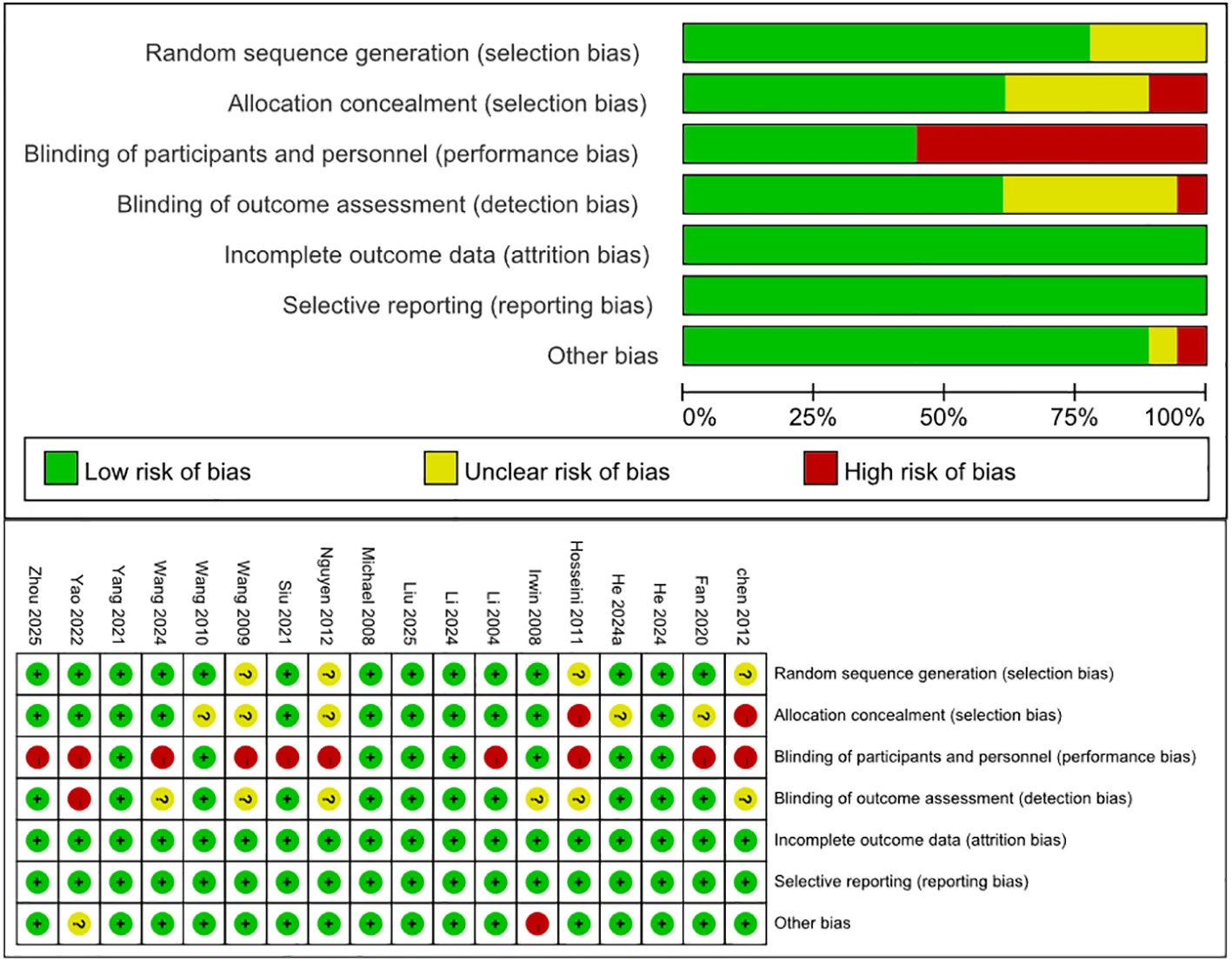
Risk assessment result.

### 3.4 Direct pairwise meta-analyses (primary outcome)

This study systematically evaluated the effects of three interventions—TC, BDJ, and TCCWTMS—on sleep quality improvement by synthesizing multiple randomized controlled trials involving 1,558 participants. The sleep quality scores are rated on a scale where lower scores indicate better outcomes. **Figure 3** presents the results of the forest plot analysis, which compares each intervention with the control group. In the direct pairwise meta-analysis, the pooled effect size for the TC group was SMD = −0.73 (95% CI: −1.21, −0.25, P = 0.003), indicating a significant improvement, but with high heterogeneity (I² = 92%), suggesting substantial differences among studies in terms of intervention protocols, research methods, or study populations. The pooled effect size for CON versus BDJ was SMD = -0.91 (95% CI: -1.59, -0.23, P = 0.009), also showing a significant effect, but with high heterogeneity (I² = 87%). The effect size for CON versus TCCWTMS was SMD = -1.14 (95% CI: -2.01, -0.27, P = 0.01), showing the largest effect size, but there was still high heterogeneity (I² = 86%). The overall pooled effect size was SMD = −0.82 (95% CI: −1.17, −0.48, P < 0.0001), with an overall heterogeneity of I² = 90%. Tests for subgroup differences were not statistically significant, indicating that there was no significant difference in overall efficacy among the three intervention methods. In summary, the available evidence suggests that TC, BDJ, and TCCWTMS can all effectively improve sleep quality, with Tai Chi combined with transcranial magnetic stimulation showing the greatest potential for effect.

**Figure 3:**
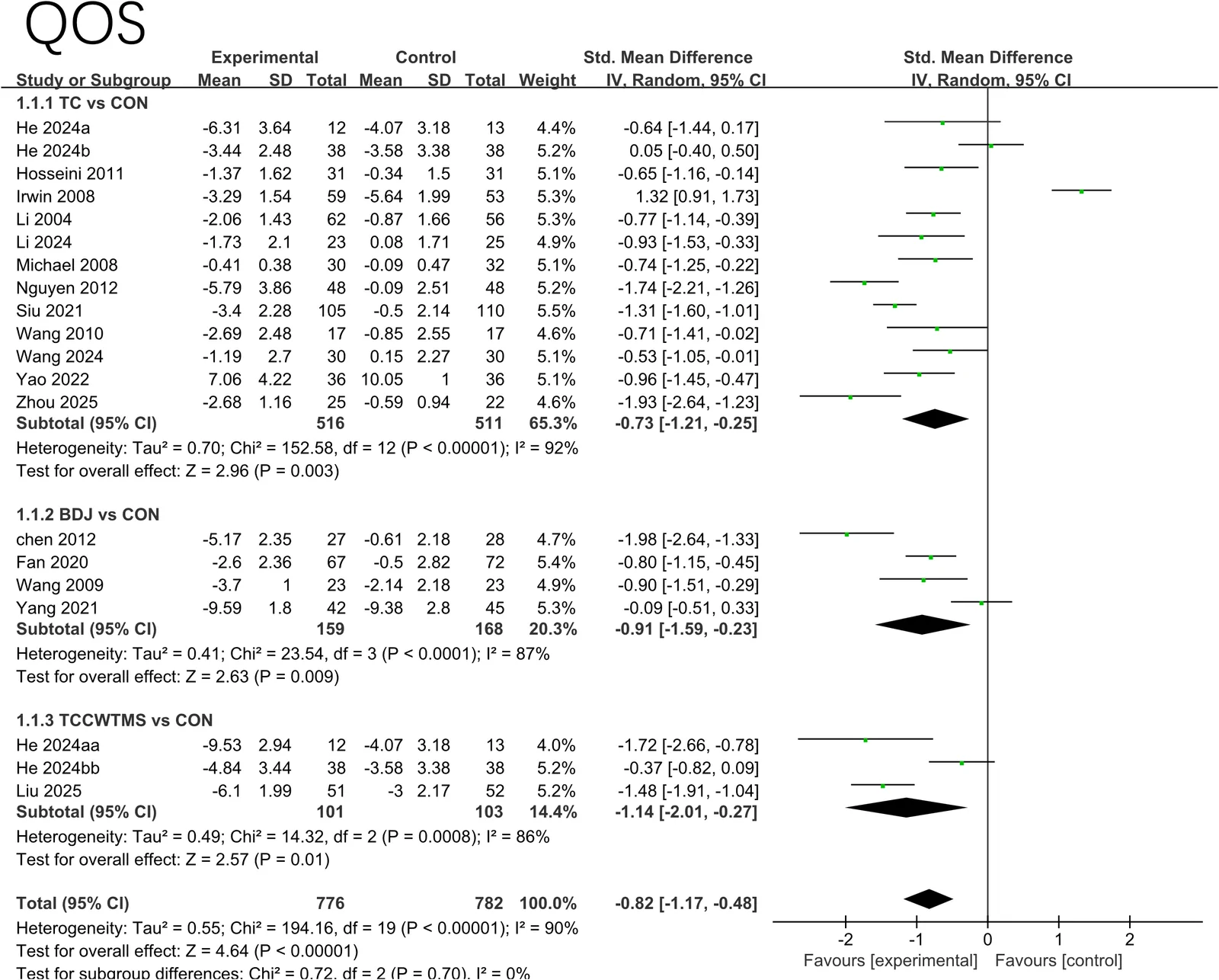
Forest of quality of sleep.

With regard to improvements in MH, a total of 755 participants were involved; a higher score indicates better mental health. **Figure 4** presents the results of the forest plot analysis, which compares each intervention with the control group. In the direct pairwise meta-analysis, the pooled effect size for the TC group was SMD = 0.50 (95% CI: 0.01, 0.99, P = 0.05), indicating a significant improvement, but with high heterogeneity (I² = 77%), suggesting substantial differences among studies in terms of intervention protocols, research methods, or study populations. The pooled effect size for BDJ versus CON was SMD = 1.30 (95% CI: -0.12, 2.72, P = 0.07), which did not reach statistical significance, and heterogeneity was extremely high (I² = 95%). The effect size for TCCWTMS versus CON was SMD = 0.54 (95% CI: -0.43, 1.50, P = 0.28), which also did not reach statistical significance and exhibited high heterogeneity (I² = 90%). The overall pooled effect size was SMD = 0.76 (95% CI: 0.29, 1.22, P = 0.002), with an overall heterogeneity of I² = 87%. Due to the high heterogeneity observed in both the subgroups and the overall analysis, the effects varied significantly across studies and should be interpreted with caution. See **Figure 4**.

**Figure 4:**
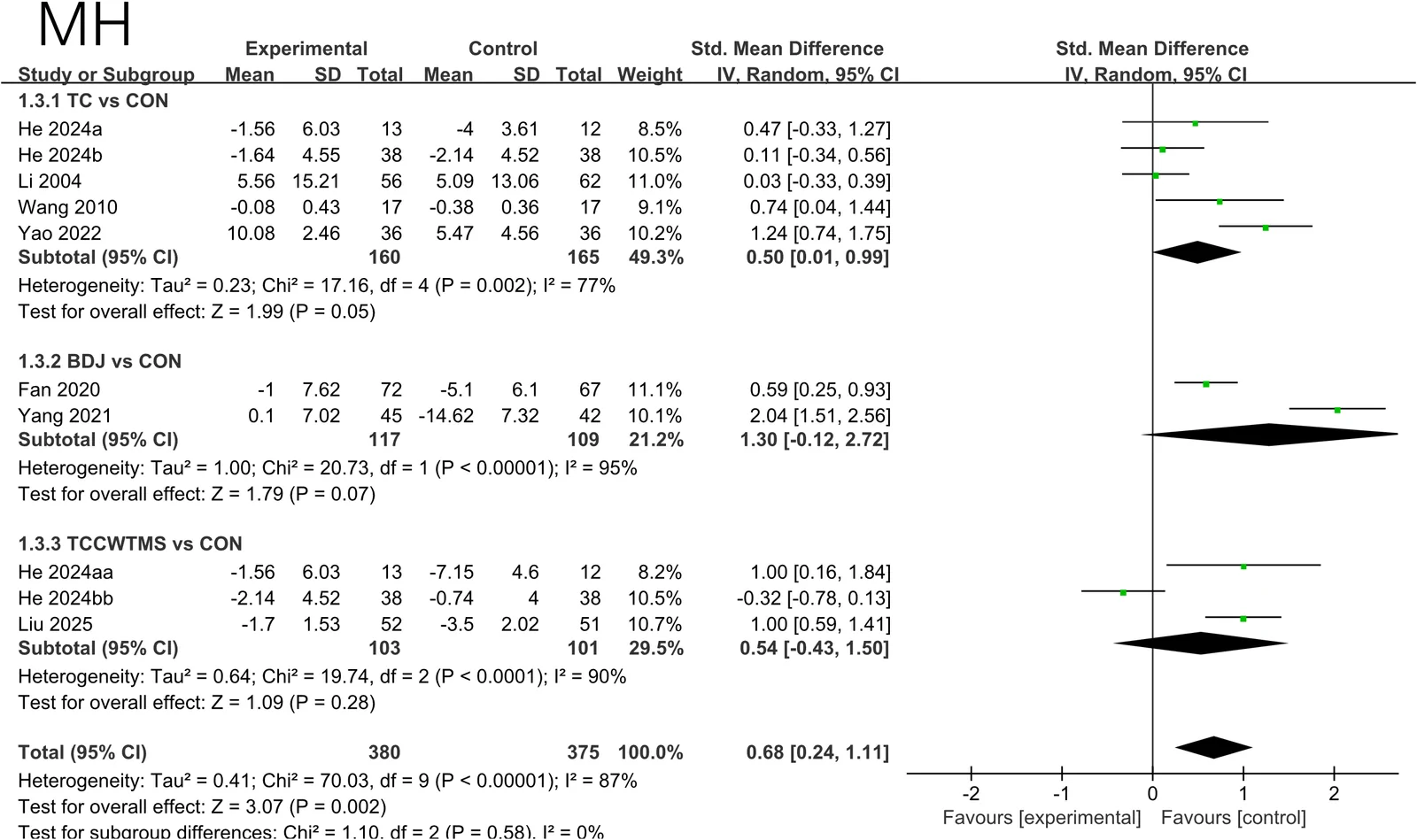
Forest of mental health.

With regard to QOL, this study compiled data from 634 participants. Lower scores indicate better outcomes. **Figure 5** presents the results of the forest plot analysis, showing that the pooled effect size for the TC group was SMD = −0.47 (95% CI: −0.71, −0.24, P < 0.0001), indicating a significant improvement, with extremely low heterogeneity (I² = 0%), suggesting a high degree of consistency among the studies. The pooled effect size for the BDJ group was SMD = -0.57 (95% CI: -0.91, -0.23, P = 0.001), also showing a significant effect; however, as only the single study by Fan (2020) was included, heterogeneity was not applicable. The effect size for the TCCWTMS group was SMD = 0.26 (95% CI: -0.72, 1.24, P = 0.60), which did not reach statistical significance, and there was high heterogeneity (I² = 90%), indicating that the effectiveness of this intervention varied considerably across different studies. The overall pooled effect size was SMD = −0.24 (95% CI: −0.73, 0.26, P = 0.35), which did not reach statistical significance, with an overall heterogeneity of I² = 89%. In summary, the available evidence suggests that both TC and BDJ can significantly improve quality of life, and the results of studies on Tai Chi are highly consistent; however, TCCWTMS did not show statistically significant improvements, and with high heterogeneity among studies, its efficacy remains inconsistent. See **Figure 5**. The results of the subgroup analysis and meta-regression are provided in the **Supplementary Materials**.

**Figure 5:**
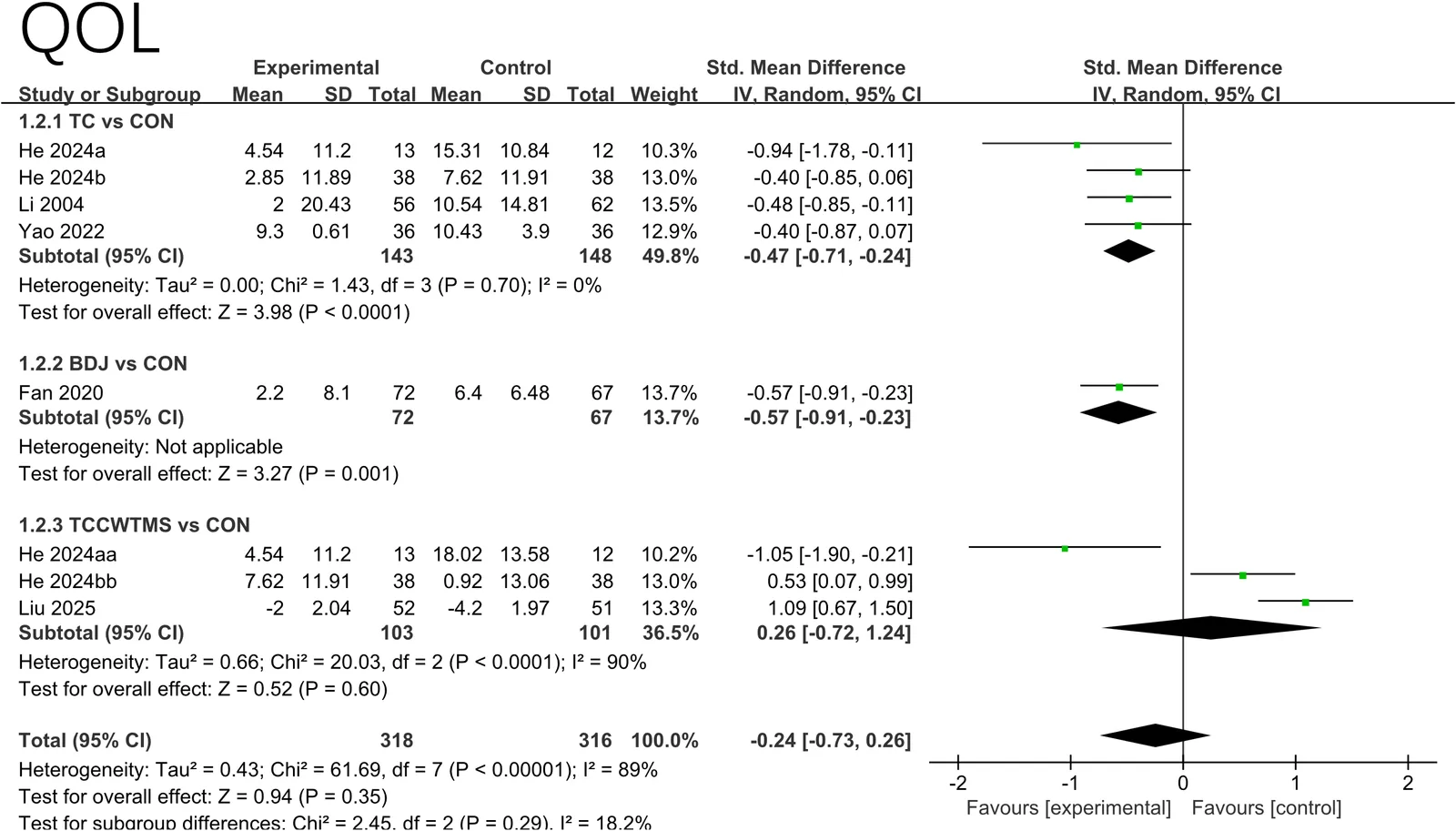
Forest of quality of life.

Sensitivity analyses confirmed the robustness of our findings; pooled estimates and 95% CIs remained stable across all outcomes when any single study was excluded, indicating no single study disproportionately drove the overall results. Detailed results are provided in the **Supplementary Materials**.

### 3.5 Network meta-analysis

**Figure 6** presents a network diagram of a meta-analysis of three traditional Chinese mind-body therapies to evaluate the efficacy of these three interventions. Node size reflects the sample size for each therapy, while line thickness indicates the number of studies comparing these treatments. Among them, TC is the most commonly used method, while TCCWTMS has fewer associated studies. **Figure 6** provides a detailed network diagram of the outcome measures.

**Figure 6:**
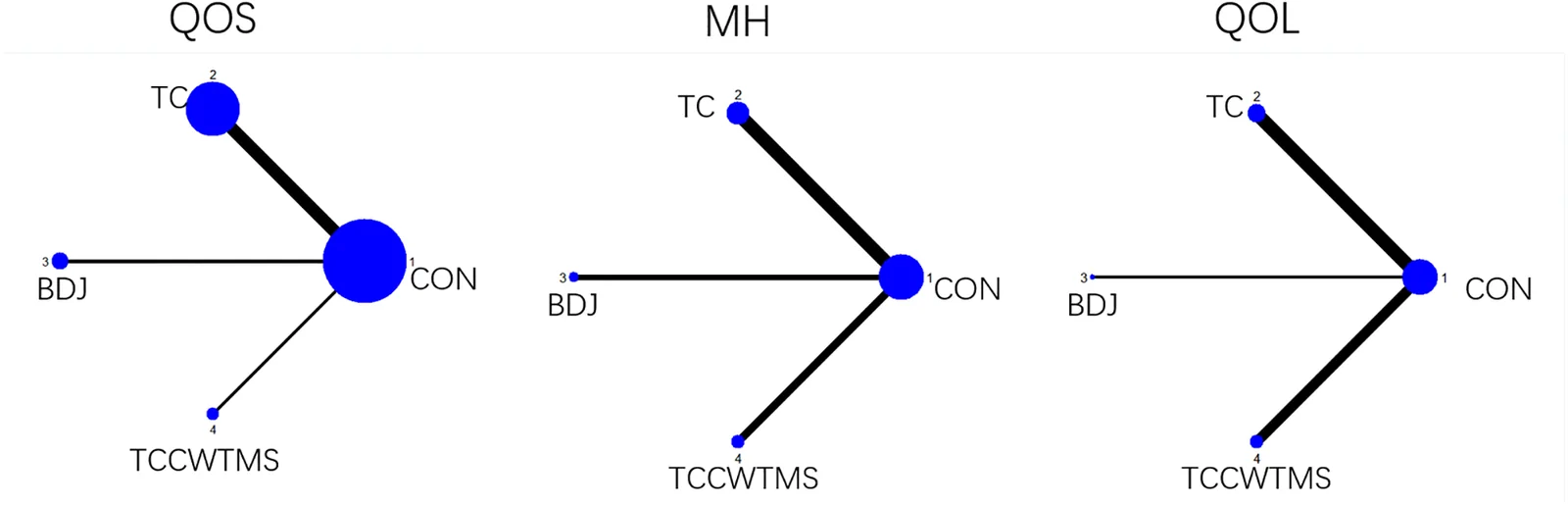
Network plot of outcome indicators.

### 3.6 Ranking of the effectiveness of three exercise interventions

The results of the network meta-analysis showed differences in the ranking of efficacy among the three interventions aimed at improving QOS. According to the SUCRA score, TCCWTMS ranked highest in terms of efficacy, with a SUCRA value of 81.2% and a Hedges’ g of -1.15 (95% CI: -2.07 to -0.22). BDJ ranked second, with a SUCRA value of 66.4% and a Hedges’ g of -0.92 (95% CI: -1.70 to -0.13). TC ranked third, with a SUCRA value of 51.7% and a Hedges’ g of -0.72 (95% CI: -1.16 to -0.29).

In terms of improving MH, BDJ ranked highest, with a SUCRA value of 92.6% and a Hedges’ g of 1.28 (95% CI: 0.30, 2.26). TCCWTMS ranked second, with a SUCRA value of 52.3% and a Hedges’ g of 0.53 (95% CI: -0.30, 1.36). TC ranked third, with a SUCRA of 48.4% and a Hedges’ g of 0.46 (95% CI: -0.18, 1.10).

In terms of improving QOL, BDJ ranked highest, with a SUCRA value of 78.8% and a Hedges’ g of -0.55 (95% CI: -1.51, 0.41). TC ranked second, with a SUCRA value of 70.8% and a Hedges’ g of -0.44 (95% CI: -1.38, 0.50). TCCWTMS ranked third, with a SUCRA value of 14.9% and a Hedges’ g of -0.30 (95% CI: -1.11, 0.52). See **Figures 7** and **8**.

**Figure 7:**
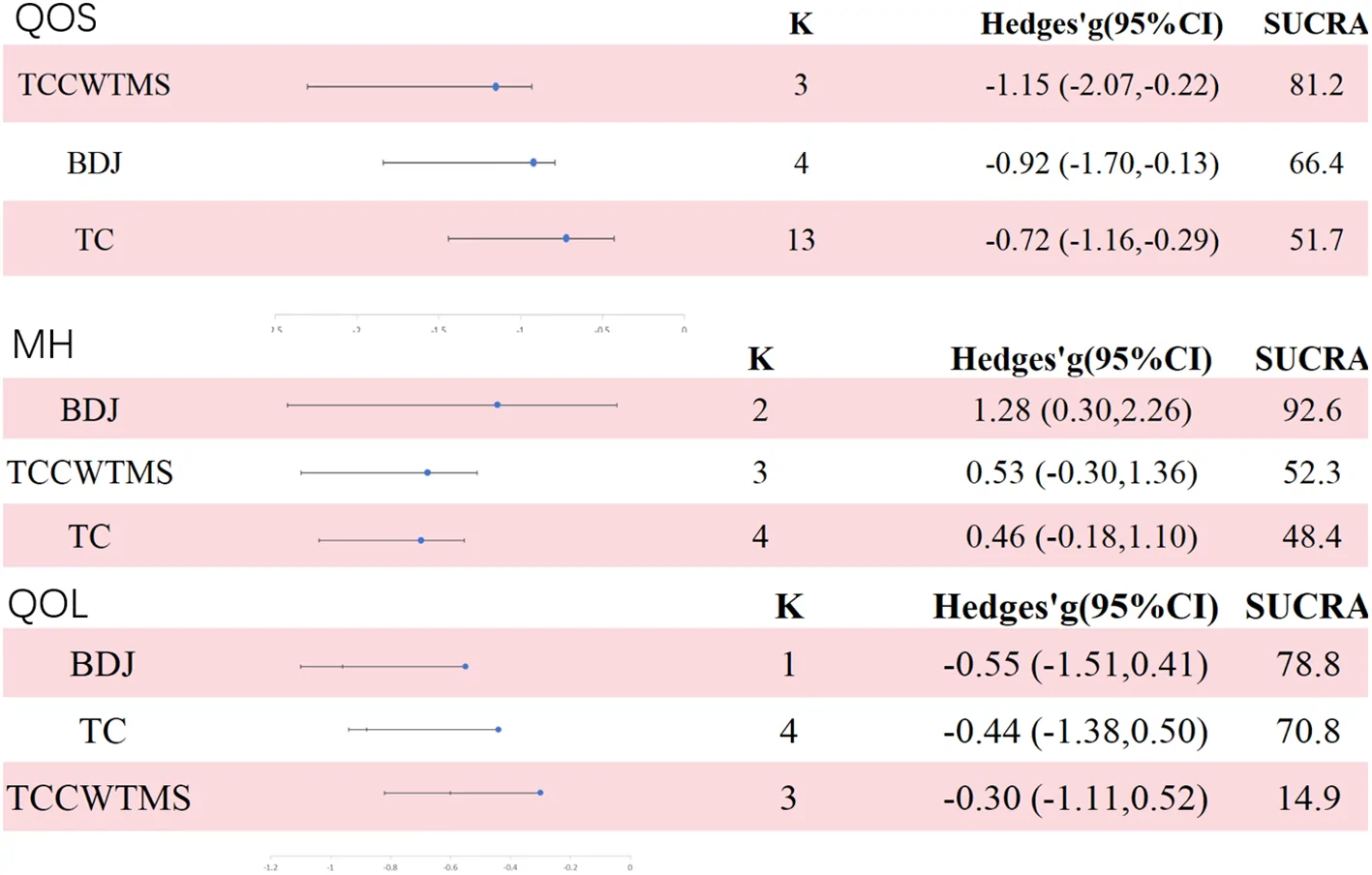
Summary of SUCRA rankings for various intervention.

**Figure 8:**
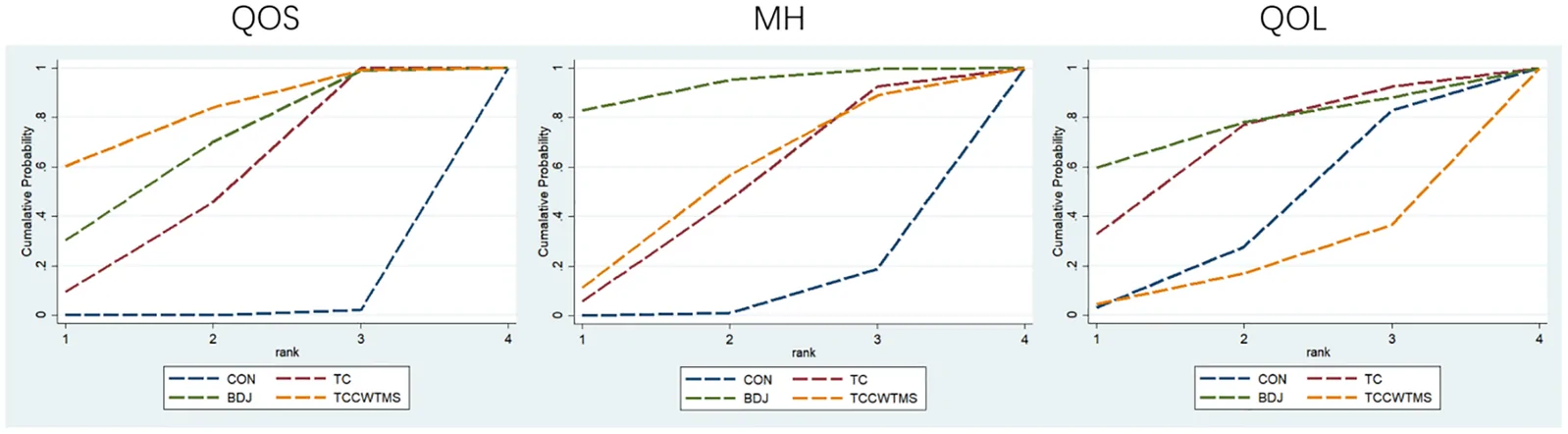
Ranking of intervention effects for outcome indicators.

### 3.7 Small sample effect or publication bias tests

For the studies included in the network meta-analysis, we used adjusted comparison funnel plots to visually assess potential small-sample effects and publication bias across the three outcome domains. However, due to the limited number of studies involved in certain direct comparisons (e.g., the tccwtms group included only three studies), the statistical power of the funnel plot asymmetry test was inherently low. **Figure 9** shows that the effect sizes for most comparisons exhibit a relatively symmetrical distribution; however, given the sparse data at several nodes in the network, it is still not possible to reliably rule out the influence of publication bias and small-sample effects. Therefore, these results should be interpreted with caution. See **Figure 9**.

**Figure 9:**
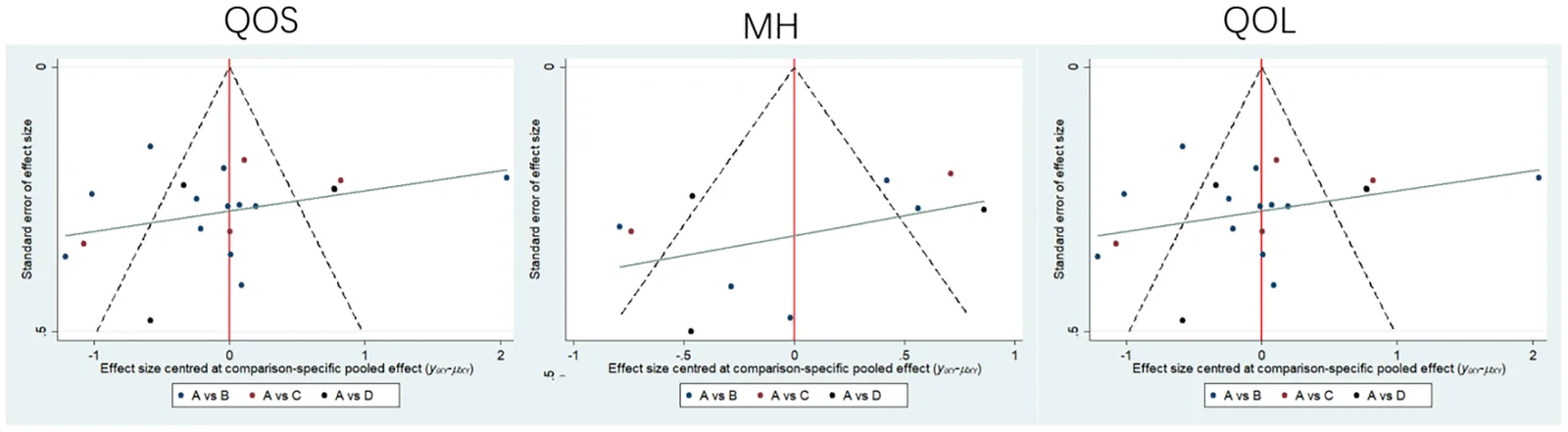
Corrected comparison funnel plot for outcome indicators. **(A)**=CON; **(B)** =TC; **(C)** =BDJ; **(D)** =TCCWTMS.

### 3.8 GRADE assessment

The GRADE assessment rated the certainty of evidence as low for all three outcomes (sleep quality, mental health, and quality of life), primarily due to serious concerns regarding risk of bias and imprecision. No serious inconsistency or indirectness was detected. See **Figure 10**.

**Figure 10:**
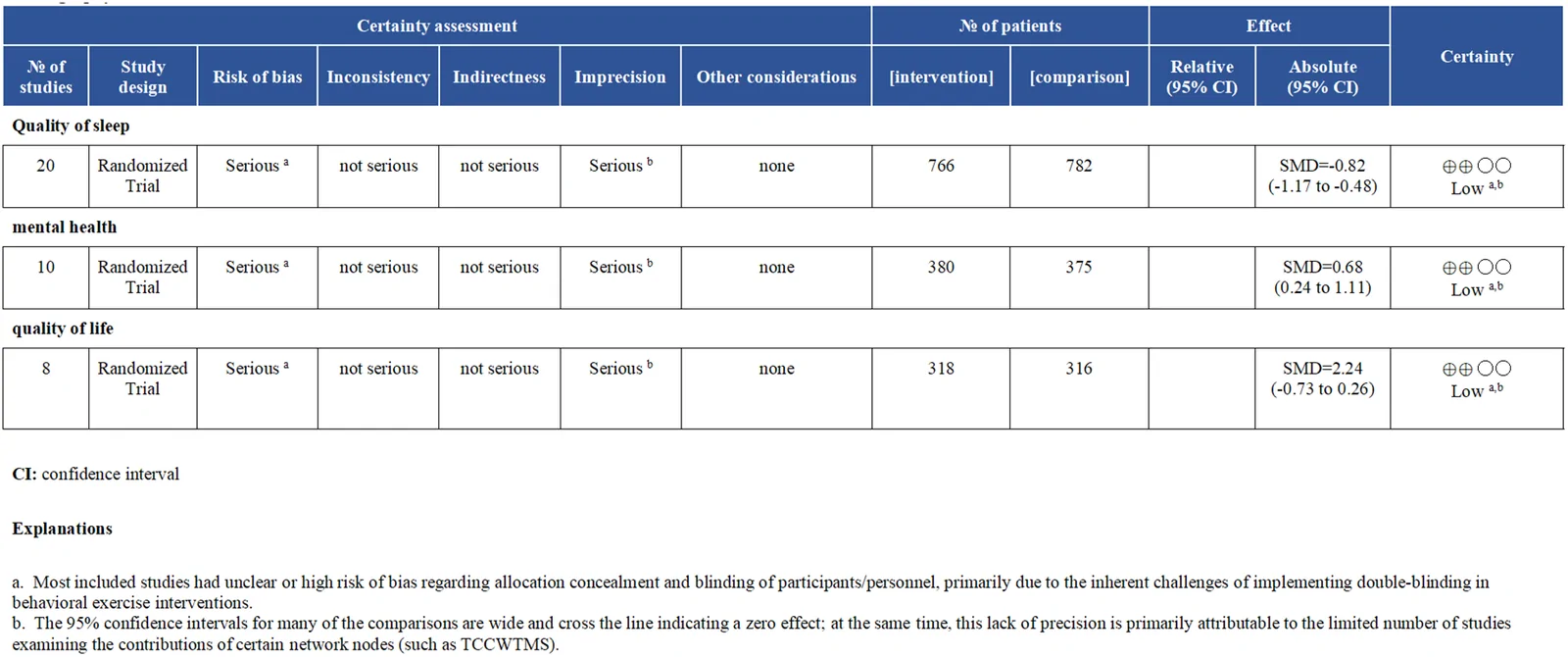
GRADE assessment.

## 4 Discussion

This study conducted a network meta-analysis incorporating 18 randomized controlled trials to systematically evaluate the effects of three traditional Chinese mind-body exercise therapies—TC, BDJ, and TCCWTMS—on sleep quality, mental health, and quality of life in patients with sleep disorders. The overall results clearly support the positive effects of all three interventions in improving the three core outcome measures (20, 21), and significant hierarchical differences in efficacy were observed among the different intervention modalities (22). This largely achieved the core objective of this study: to identify the optimal traditional Chinese mind-body exercise therapy for each outcome measure (23).

This study found that TC, BDJ, and TCCWTMS all significantly improved sleep quality in patients with sleep disorders, with TCCWTMS showing the most pronounced efficacy, followed by BDJ, while TC demonstrated relatively limited effects. These results suggest that TCCWTMS may produce a synergistic effect with the mind-body integration training of Tai Chi by regulating cortical excitability and restoring the neural circuits of the sleep-wake cycle, thereby more effectively improving sleep (24, 25). This is consistent with the pathological characteristic of heightened central nervous system arousal commonly observed in patients with sleep disorders (24, 26). Compared with previous studies that focused solely on single mind-body exercises or conventional treatments, this network meta-analysis confirms that combined interventions provide greater sleep benefits for patients (26), and that there are clear differences in efficacy levels among different monotherapies (27). However, due to the small number of studies included in the combined intervention group, there was heterogeneity across studies regarding intervention frequency, duration, and sleep measurement tools.

Regarding improvements in the mental health of patients with sleep disorders, only the TC intervention demonstrated statistically significant improvements, while neither BDJ nor TCCWTMS reached statistical significance. However, the SUCRA rankings suggested that BDJ may hold the highest potential, followed by TCCWTMS, with TC ranking lowest. This seemingly contradictory observation—where BDJ ranked highest but failed to achieve statistical significance—suggests that the evidence base in this field remains insufficient, with high heterogeneity among studies leading to imprecise effect estimates (28). Compared to previous studies concluding that BDJ significantly improves mental health (29), this study failed to fully replicate this finding, possibly due to small sample sizes in the included studies and inconsistencies in the frequency and duration of the intervention protocols. It is noteworthy that although the TC intervention achieved statistical significance in pairwise comparisons, it ranked last in the SUCRA ranking. This suggests that while the efficacy of Tai Chi is consistently demonstrated in some studies, its potential effect size is probably smaller compared to other interventions (30). Overall, the current evidence is insufficient to definitively determine which intervention is most effective in improving mental health. Given the scarcity of direct head-to-head evidence, the existing ranking results should be interpreted with caution and viewed primarily as a preliminary reference for clinical reasoning rather than a conclusive endorsement (31).

In terms of improving QOL, both TC and BDJ demonstrated statistically significant improvements, and the results of TC-related studies were highly consistent (32); in contrast, TCCWTMS did not reach statistical significance. The SUCRA-based probability rankings suggested a potential advantage of BDJ over TC, while TCCWTMS appeared to rank lowest. These results indicate that traditional mind-body exercise therapy alone offers clear and consistent advantages in improving patients’ quality of life, whereas the addition of transcranial magnetic stimulation did not provide additional benefits. Instead, it may have failed to demonstrate positive effects due to factors such as increased treatment burden or insufficient sample size. As low-intensity, highly accessible mind-body integrated training programs, BDJ and TC can be better integrated into patients’ daily lives, thereby producing lasting improvements in quality of life (33). This study provides further insight into the differential efficacy of these interventions on QOL, suggesting that, based on the currently limited direct evidence, combined therapy may not be superior to single-modality mind-body exercise for this outcome measure (25). However, it should be noted that the number of studies included in the combined intervention group was small and heterogeneity was high, making its efficacy inconsistent; the current evidence is insufficient to completely rule out its potential value (34).

In summary, this study used a network meta-analysis to compare the relative efficacy of three interventions for patients with sleep disorders. Unlike existing reviews, which primarily focus on paired comparisons of single therapies, this study provides a quantitative ranking of efficacy. The practical implications are as follows: patients primarily concerned with sleep disorders may prioritize combination therapies; for patients who need to improve both their mental health and daily functioning, BDJ or TC are preferable options due to their low cost and ease of access. This study also points the way for future research using larger sample sizes and standardized intervention protocols.

### 4.1 Limitation

Several limitations are worth considering. First, the risk of bias regarding allocation concealment and blinding was mostly rated as “unclear” or “high.” Although this is an inherent characteristic of exercise-based behavioral interventions, the lack of blinding may have introduced performance and detection biases. Second, the relatively small number of studies for certain comparisons (e.g., TCCWTMS) resulted in high statistical heterogeneity and wide confidence intervals. These limitations underscore the need for cautious interpretation of the current findings and highlight the importance of conducting large-scale, strictly blinded randomized controlled trials (RCTs) in the future.

## 5 Conclusion

This study found that all three interventions demonstrated significant results in improving QOS, with TCCWTMS showing the best efficacy. Regarding improvements in MH, the current evidence is insufficient to identify the optimal intervention; although BDJ ranked first, the results were not statistically significant. Only the TC intervention showed significant effects, but the overall evidence remains insufficient. In terms of improving QOL, BDJ demonstrated the best efficacy, while TCCWTMS did not show significant effects. This study provides evidence-based guidance for clinical decision-making in patients with sleep disorders. For patients whose primary concern is sleep disturbance, combined therapy may be prioritized, while patients seeking to improve their quality of life may opt for BDJ or TC. Future research should focus on standardizing intervention protocols and assessment criteria to further clarify the optimal treatment parameters and underlying neurophysiological mechanisms of different intervention strategies, thereby providing a more robust evidence base for clinical implementation.

## Data Availability

The original contributions presented in the study are included in the article/**Supplementary Material**. Further inquiries can be directed to the corresponding author.
