# Supplementary material for: A comparative study of traditional Chinese mind-body therapies on sleep quality, mental health, and quality of life in patients with sleep disorders: a network meta-analysis based on randomized controlled trials
Source: Front Psychiatry. 2026 Sep 18;17:1921194. doi: 10.3389/fpsyt.2026.1921194 (PMC13631947; doi:10.3389/fpsyt.2026.1921194)
Supplement: Supplementary file 1 [file SupplementaryFile1.docx]

**Supplementary Materials2 Search strategy**

## Search strategy of PubMed

Search Terms

("Sleep Initiation and Maintenance Disorders"[MeSH Terms] OR "Sleep Wake Disorders"[MeSH Terms] OR "Insomnia"[MeSH Terms] OR "Chronobiology Disorders"[MeSH Terms] OR "sleep disorder*"[Title/Abstract] OR "insomnia*"[Title/Abstract] OR "sleep disturbance*"[Title/Abstract] OR "sleep quality"[Title/Abstract] OR "poor sleep"[Title/Abstract] OR "PSQI"[Title/Abstract] OR "Pittsburgh Sleep Quality Index"[Title/Abstract] OR "sleep efficiency"[Title/Abstract] OR "sleep latency"[Title/Abstract])

AND

("Tai Ji"[MeSH Terms] OR "Tai Chi"[Title/Abstract] OR "Taijiquan"[Title/Abstract] OR "Baduanjin"[Title/Abstract] OR "Eight-Section Brocade"[Title/Abstract] OR "Qigong"[MeSH Terms] OR "Qigong"[Title/Abstract] OR "Chi Kung"[Title/Abstract] OR "Mind-Body Therapies"[MeSH Terms] OR "mind body exercise*"[Title/Abstract] OR "traditional Chinese exercise*"[Title/Abstract] OR "traditional Chinese mind body"[Title/Abstract] OR "transcranial magnetic stimulation"[MeSH Terms] OR "transcranial magnetic stimulation"[Title/Abstract] OR "TMS"[Title/Abstract] OR "rTMS"[Title/Abstract] OR "repetitive transcranial magnetic stimulation"[Title/Abstract] OR "non-invasive brain stimulation"[Title/Abstract] OR "NIBS"[Title/Abstract] OR "neuromodulation"[Title/Abstract])

AND

("Mental Health"[MeSH Terms] OR "mental health"[Title/Abstract] OR "psychological well-being"[Title/Abstract] OR "depression"[MeSH Terms] OR "depression"[Title/Abstract] OR "depressive symptom*"[Title/Abstract] OR "anxiety"[MeSH Terms] OR "anxiety"[Title/Abstract] OR "anxiety symptom*"[Title/Abstract] OR "Quality of Life"[MeSH Terms] OR "quality of life"[Title/Abstract] OR "QoL"[Title/Abstract] OR "health-related quality of life"[Title/Abstract] OR "HRQOL"[Title/Abstract] OR "well-being"[Title/Abstract])

AND

("Randomized Controlled Trial"[Publication Type] OR "Controlled Clinical Trial"[Publication Type] OR "randomized"[Title/Abstract] OR "randomised"[Title/Abstract] OR "RCT"[Title/Abstract] OR "random allocation"[Title/Abstract])

**Supplementary Materials3**

Table 1. Basic features of the summary

| Study | Country | Duration of intervention | Group | Sample(M/F) | Age (mean±SD) | illness duration | Intervention | Frequency of intervention | Outcome measures |
| --- | --- | --- | --- | --- | --- | --- | --- | --- | --- |
| chen 2012[1] | Taiwan | 12 weeks | baduanjin | 27 (10/17) | 70.48 ± 7.90 | no report | Baduanjin exercise | 3 times/week，40min/time | PSQI |
|  |  |  | Con | 28 (9/19) | 72.96 ± 8.30 | no report | no intervention |  |  |
| Hosseini 2011[2] | Iran | 12 weeks | Tai Chi | 31 (16/15) | 68.74 ± 5.48 | no report | Tai Chi | 3 times/week, 5-25 min/time | PSQI |
|  |  |  | Con | 31 (14/17) | 69.42 ± 5.34 | no report | no intervention |  |  |
| Yao 2022[3] | China | 8 weeks | Tai Chi | 36 (0/36) | 45.3 ± 8.5 | no report | Tai Chi | 2 times/week, 60 min/session | PSQI, HADS-D, FACT-B |
|  |  |  | Con | 36 (0/36) | 48.6 ± 7.8 | no report | Routine care |  |  |
| Liu 2025[4] | China | 6 weeks | taichi+ rTMS） | 55 (18/37) | 67.8 ± 4.5 | no report | taich + 1-Hz rTMS | taichi：5times/week，60/time；rTMS：5time/week，20min/time | PSQI, MoCA, HAMA, HAMD, ESS |
|  |  |  | con + shamrTMS | 55 (24/31) | 68.0 ± 4.6 | no report | con + sham rTMS | taichi：5times/week，60/time；rTMS：5time/week，20min/time |  |
| Irwin 2008[5] | USA | 25 weeks | Tai Chi | 59 (41/18) | 69.7 ± 6.1 | no report | Tai Chi | 3 times/week, 40 min/session | PSQI |
|  |  |  | Con | 53 (30/23) | 70.3 ± 7.6 | no report | Health Education | 3 times/week, 40 min/session |  |
| Nguyen 2012[6] | Vietnam | 6 months | Tai Chi | 48 | 69.2 ± 5.3 | no report | 24-form Tai Chi | 2 times/week, 60 min/session | PSQI |
|  |  |  | Con | 48 | 68.7 ± 4.9 | no report | routine activities |  |  |
| Siu 2021[7] | Hong Kong | 12 weeks | Tai Chi | 105 (21/84) | 67.3 ± 6.8 | 124.4 ± 134.5 months | Tai Chi | 3 times/week, 60 min/session | PSQI |
|  |  |  | Con | 110 (22/88) | 67.3 ± 6.8 | 124.4 ± 134.5 months | usual care |  |  |
| Wang 2009[8] | China | 4 months | Baduanjin | 23 | 57.8 | DM: 89.04 ± 72.21 Month | Baduanjin | no report | PSQI |
|  |  |  |  |  |  | Insomnia: 40.85 |  |  |  |
|  |  |  | Control | no report | 57.8 | DM: 89.04 ± 72.21Month | Routine care | no report |  |
|  |  |  |  |  |  | Insomnia: 40.85 |  |  |  |
| Zhou 2025[9] | China | 24 weeks | Tai Chi | 25 (4/21) | 19.24 ± 0.66 | no report | Tai Chi | 3 times/week, 60 min/session | PSQI |
|  |  |  | Control | 22 (7/15) | 18.86 ± 0.64 | no report | usual lifestyle |  |  |
| Li 2004[10] | USA | 24 weeks | Tai Chi | 62 (10/52) | 75.30 ± 7.8 | no report | Tai Chi | 3 times/week, 60 min/session | PSQI, ESS, SF-12 |
|  |  |  | Con | 56 (12/44) | 75.45 ± 7.8 | no report | Seated stretching + controlled breathing + relaxation | 3 times/week, 60 min/session |  |
| Wang 2024[11] | China | 8 weeks | Tai Chi | 30 (4/26) | 66.0 ± 4.97 | no report | Tai Chi | 3 times/week, 60 min/session | PSQI, ISI, ESS, PSG |
|  |  |  | Con | 30 (4/26) | 69.1 ± 4.56 | no report | Maintain normal daily routines |  |  |
| He 2024[12] | China | 4w | Tai Chi | 12（3/9） | 68.17 ± 4.47 | no report | Tai Chi | 60min,3day/week | Sleep efficiency |
|  |  |  | con | 13(2/11) | 69.85 ± 5.37 | no report | Health Education | 60min,3day/week |  |
| He 2024a[13] | China | 4w | taichi+ rTMS） | 38（12/26） | 66.63 ± 5.32 | no report | taich + rTMS | 5 times/week, 30 min/session | ISI;Sleep efficiency; Sleep onset latency |
|  |  |  | con | 38(11/27) | 68.50 ± 4.97 | no report | con | 3 times/week, 60 min/session |  |
| Li 2024[14] | China | 8w | con | 25（4/21） | 68.92±4.64 | no report | Tai Chi | 60min,3day/week | ISI (0-28); PSQI (0-21) |
|  |  |  | Tai Chi | 23(2/21) | 66.13±4.29 | no report | con |  |  |
| Michael 2008[15] | USA | 25w | Tai Chi | 29(10/19) | 69.6 ±6.3 | no report | Tai Chi | 40min,3day/week | Global Sleep Quality; Sleep Quality |
|  |  |  | health education | 30(8/22) | 69.7 ±6.1 | no report | con | 40min,3day/week |  |
| Wang 2010[16] | Japan. | 12w | routine care | 17（5/12） | 77.59 ± 12.33 | no report | routine care | 80min,1day/week | Global sleep quality; Sleep quality |
|  |  |  | Tai Chi | 17(4/13) | 76.53 ± 9.74 | no report | Tai Chi | 80min,1day/week |  |
| Fan 2020[17] | China | 24w | routine care | 72（22/50） | 71.8 ± 6.7 | no report | routine care | 45min,5day/week | PSQI; SF-36 |
|  |  |  | daduajin | 67(12/55) | 70.3 ± 5.7 | no report | daduajin | 45min,5day/week |  |
| Yang 2021[18] | China | 12 days | dadfuanjin | 42（22/20） | 45±11 | no report | dadfuanjin | 7 times/weeks | PSQI |
|  |  |  | routine care | 45(21/24) | 45±11 | no report | routine care | 7 times/weeks |  |

**Supplementary Materials 4**

Table 2. Evaluation results of literature quality risk bias of included studies

| Study | Randomization Methods | Hidden Allocation Schemes | Blinding | Blinding in Outcome Assessment | Completeness of Data | Selective Reporting of Studies | Other Sources of Bias (Small Sample Size, Baseline Imbalance) |
| --- | --- | --- | --- | --- | --- | --- | --- |
| chen 2012 | unclear | high risk | high risk | unclear | low risk | low risk | low risk |
| Hosseini 2011 | unclear | high risk | high risk | unclear | low risk | low risk | low risk |
| Yao 2022 | low risk | low risk | high risk | high risk | low risk | low risk | unclear |
| Liu 2025 | low risk | low risk | low risk | low risk | low risk | low risk | low risk |
| Irwin 2008 | low risk | low risk | low risk | low risk | low risk | low risk | high risk |
| Nguyen 2012 | unclear | unclear | high risk | unclear | low risk | low risk | low risk |
| Siu 2021 | low risk | low risk | high risk | low risk | low risk | low risk | low risk |
| Wang 2009 | unclear | unclear | high risk | unclear | low risk | low risk | low risk |
| Zhou 2025 | low risk | low risk | high risk | low risk | low risk | low risk | low risk |
| Li 2004 | low risk | low risk | high risk | low risk | low risk | low risk | low risk |
| Wang 2024 | low risk | low risk | high risk | unclear | low risk | low risk | low risk |
| He 2024 | low risk | low risk | low risk | low risk | low risk | low risk | low risk |
| He 2024a | low risk | unclear | low risk | low risk | low risk | low risk | low risk |
| Li 2024 | low risk | low risk | low risk | low risk | low risk | low risk | low risk |
| Michael 2008 | low risk | low risk | low risk | low risk | low risk | low risk | low risk |
| Wang 2010 | low risk | unclear | low risk | low risk | low risk | low risk | low risk |
| Fan 2020 | low risk | unclear | high risk | low risk | low risk | low risk | low risk |
| Yang 2021 | low risk | low risk | low risk | low risk | low risk | low risk | low risk |

**Supplementary Materials 5 Subgroup analysis**

MH Subgroup analysis (by age)

MH Subgroup analysis (by week)

QOL Subgroup analysis (by age)

QOL Subgroup analysis (by week)

QOS Subgroup analysis (by age)

QOS Subgroup analysis (by week)

**Supplementary Materials 6 Meta Regression**

**Table 3**. Meta-regression analyses of the effects of participant mean age and intervention duration on MH, QOL, and QOS.

| MH | Covariate | Coefficient (β*β*) | Standard Error (SE) | 95% CI | t*t* value | P*P* value | Ires2*Ires*2 (%) | Adj R2*Adj* *R*2 (%) |
| --- | --- | --- | --- | --- | --- | --- | --- | --- |
|  | **Mean age** | 0.286 | 0.104 | [0.047, 0.526] | 2.76 | 0.025 | 86.64 | 45.86 |
|  | **week** | 0.570 | 0.124 | [0.283, 0.857] | 4.58 | 0.002 | 0.00 | 100.00 |
| QOL | Covariate | Coefficient (β*β*) | Standard Error (SE) | 95% CI | t*t* value | P*P* value | Ires2*Ires*2 (%) | Adj R2*Adj* *R*2 (%) |
|  | **Mean age** | 0.162 | 2.188 | [-5.190, 5.515] | 0.07 | 0.943 | 0.00 | N/A |
|  | **week** | 0.141 | 0.516 | [-1.121, 1.404] | 0.27 | 0.793 | 63.94 | -16.25 |
| QOS | Covariate | Coefficient (β*β*) | Standard Error (SE) | 95% CI | t*t* value | P*P* value | Ires2*Ires*2 (%) | Adj R2*Adj* *R*2 (%) |
|  | **Mean age** | 0.002 | 0.511 | [-1.071, 1.075] | 0.00 | 0.997 | 0.00 | N/A |
|  | **week** | -0.080 | 0.115 | [-0.322, 0.163] | -0.69 | 0.498 | 0.00 | N/A |

**Supplementary Materials 7 Sensitivity Analysis**

Mental health

m

Quality of life

Quality of sleep

**Reference**

[1] Chen, M. C., Liu, H. E., Huang, H. Y., & Chiou, A. F. (2012). The effect of a simple traditional exercise programme (Baduanjin exercise) on sleep quality of older adults: a randomized controlled trial. International journal of nursing studies, 49(3), 265–273. <https://doi.org/10.1016/j.ijnurstu.2011.09.009>
[2] Hosseini, H., Esfirizi, M. F., Marandi, S. M., & Rezaei, A. (2011). The effect of Ti Chi exercise on the sleep quality of the elderly residents in Isfahan, Sadeghieh elderly home. Iranian journal of nursing and midwifery research, 16(1), 55–60.
[3] Yao, L. Q., Kwok, S. W. H., Tan, J. B., Wang, T., Liu, X. L., Bressington, D., Chen, S. L., & Huang, H. Q. (2022). The effect of an evidence-based Tai chi intervention on the fatigue-sleep disturbance-depression symptom cluster in breast cancer patients: A preliminary randomised controlled trial. European journal of oncology nursing : the official journal of European Oncology Nursing Society, 61, 102202. <https://doi.org/10.1016/j.ejon.2022.102202>
[4] Liu, Z., Zhang, L., Bai, L., Guo, Z., Gao, J., Lin, Y., Zhou, Y., Lai, J., Tao, J., & Chen, L. (2025). Repetitive Transcranial Magnetic Stimulation and Tai Chi Chuan for Older Adults With Sleep Disorders and Mild Cognitive Impairment: A Randomized Clinical Trial. JAMA network open, 8(1), e2454307. <https://doi.org/10.1001/jamanetworkopen.2024.54307>
[5] Irwin, M. R., Olmstead, R., & Motivala, S. J. (2008). Improving sleep quality in older adults with moderate sleep complaints: A randomized controlled trial of Tai Chi Chih. Sleep, 31(7), 1001–1008.
[6] Nguyen, M. H., & Kruse, A. (2012). A randomized controlled trial of Tai chi for balance, sleep quality and cognitive performance in elderly Vietnamese. Clinical interventions in aging, 7, 185–190. <https://doi.org/10.2147/CIA.S32600>
[7] Siu, P. M., Yu, A. P., Tam, B. T., Chin, E. C., Yu, D. S., Chung, K. F., Hui, S. S., Woo, J., Fong, D. Y., Lee, P. H., Wei, G. X., & Irwin, M. R. (2021). Effects of Tai Chi or Exercise on Sleep in Older Adults With Insomnia: A Randomized Clinical Trial. JAMA network open, 4(2), e2037199. <https://doi.org/10.1001/jamanetworkopen.2020.37199>
[8] Wang, F., Wang, W. D., Zhang, R. R., Lin, Y. N., Hong, L., Zhao, Y., Ni, Q., Ishiyasutomo, Kosikawafusako, Kisitaiti, Tutuititakuya, Suzukiako, & Harukiyutak. (2009). Influences of different qigong practices on sleep quality in patients with type 2 diabetes accompanied by insomnia. Chinese Journal of Behavioral Medicine and Brain Science, 18(7), 636-639.
[9] Zhou, X., Li, F., Feng, N., Teng, Y., & Yu, Q. (2026). Effects of brisk walking and Tai Chi interventions on sleep quality in university students with insomnia. Frontiers in public health, 13, 1732579. <https://doi.org/10.3389/fpubh.2025.1732579>
[10] Li, F., Fisher, K. J., Harmer, P., Irbe, D., Tearse, R. G., & Weimer, C. (2004). Tai chi and self-rated quality of sleep and daytime sleepiness in older adults: a randomized controlled trial. Journal of the American Geriatrics Society, 52(6), 892–900. <https://doi.org/10.1111/j.1532-5415.2004.52255.x>
[11] Wang, C., Jiang, T., Li, H., Cao, G., & Zhang, G. (2024). The effects of Tai Chi exercise on sleep quality among the elderly: a study based on polysomnographic monitoring. Frontiers in neurology, 15, 1304463. <https://doi.org/10.3389/fneur.2024.1304463>
[12] He, J., Chan, S. H., Lin, J., & Tsang, H. W. (2024). Integration of tai chi and repetitive transcranial magnetic stimulation for sleep disturbances in older adults: A pilot randomized controlled trial. Sleep medicine, 122, 35–44. <https://doi.org/10.1016/j.sleep.2024.07.029>
[13] He, J., Chan, S. H. W., Chung, R. C. K., & Tsang, H. W. H. (2024). Effect of combined Tai Chi and repetitive transcranial magnetic stimulation for sleep disturbance in older adults: A randomized controlled trial. Journal of psychiatric research, 180, 281–290. <https://doi.org/10.1016/j.jpsychires.2024.10.025>
[14] Li, L. Y., Xie, X., Jiang, H. X., & Yu, J. (2024). Improving Memory Through Better Sleep in Community-Dwelling Older Adults: A Tai Chi Intervention Study. The journals of gerontology. Series B, Psychological sciences and social sciences, 79(11), gbae156. <https://doi.org/10.1093/geronb/gbae156>
[15] Michael R. Irwin, Richard Olmstead, Sarosh J. Motivala, Improving Sleep Quality in Older Adults with Moderate Sleep Complaints: A Randomized Controlled Trial of Tai Chi Chih, Sleep, Volume 31, Issue 7, July 2008, Pages 1001–1008, <https://doi.org/10.5665/sleep/31.7.1001>
[16] Wang, W., Sawada, M., Noriyama, Y., Arita, K., Ota, T., Sadamatsu, M., Kiyotou, R., Hirai, M., & Kishimoto, T. (2010). Tai Chi exercise versus rehabilitation for the elderly with cerebral vascular disorder: a single-blinded randomized controlled trial. Psychogeriatrics : the official journal of the Japanese Psychogeriatric Society, 10(3), 160–166. <https://doi.org/10.1111/j.1479-8301.2010.00334.x>
[17] Fan, B., Song, W., Zhang, J., Er, Y., Xie, B., Zhang, H., Liao, Y., Wang, C., Hu, X., Mcintyre, R., & Lee, Y. (2020). The efficacy of mind-body (Baduanjin) exercise on self-reported sleep quality and quality of life in elderly subjects with sleep disturbances: a randomized controlled trial. Sleep & breathing = Schlaf & Atmung, 24(2), 695–701. <https://doi.org/10.1007/s11325-019-01999-w>
[18] Yang, C., Ma, Y., Mei, J. H., Gong, X., Wang, M., & Liu, K. (2021). Zhongguo zhen jiu = Chinese acupuncture & moxibustion, 41(3), 243–246. https://doi.org/10.13703/j.0255-2930.20200327-0002
